# Cognitive and neurobehavioral phenotypes of post 9/11 veterans with epilepsy and mild traumatic brain injury

**DOI:** 10.3389/fneur.2025.1706223

**Published:** 2026-01-26

**Authors:** Samin Panahi, Eamonn Kennedy, Jamie Mayo, Lee Christensen, Sreekanth Kamineni, Hari Krishna Raju Sagiraju, Tyler Cooper, Shirin Saleh, Justin M. Losciale, Angela Peters, Randall Rupper, Mary Jo Pugh

**Affiliations:** 1 VA Salt Lake City Health Care System, Informatics, Decision-Enhancement and Analytic Sciences Center, Salt Lake City, UT, United States; 2 Division of Epidemiology, University of Utah School of Medicine, Salt Lake City, UT, United States; 3 Preventive Oncology, All India Institute of Medical Sciences, New Delhi, India; 4 Department of Neurology, University of Utah School of Medicine, Salt Lake City, UT, United States; 5 VA Salt Lake City Health Care System, Geriatric Research, Education and Clinical Center, Salt Lake City, UT, United States

**Keywords:** cognitive, epilepsy, mild traumatic brain injury, military health, natural language processing, phenotyping, veterans

## Abstract

**Introduction:**

Traumatic brain injury (TBI) and epilepsy are significant health concerns among the veteran population, but the links between mild TBI and cognitive and behavioral changes in epilepsy have been little explored. This study leveraged natural language processing of medical records and chart review to assess the prevalence and patterns of cognitive and behavioral symptoms in post-9/11 veterans with epilepsy, with and without history of mild TBI. The study objective was to identify distinct neurobehavioral phenotypes, and then explore their socio-demographic factors, comorbidities, and phenotypes.

**Methods:**

We conducted a detailed chart review using NLP to extract cognitive dysfunction indicators that were categorized into seven Research Domain Criteria domains. Employing Uniform Manifold Approximation and Projection for clustering and dimensionality reduction.

**Results:**

By clustering individuals on behavioral and cognitive concepts in medical notes, this study extends beyond traditional diagnostic classifications, revealing a cognitive and behavioral phenotype of veterans. Veterans with post traumatic epilepsy often demonstrate significant cognitive risk profiles associated with RDoC domains, particularly in domains related to cognitive function and arousal/regulatory systems. Both veterans with TBI before Epilepsy post traumatic epilepsy and those with epilepsy preceding TBI displayed greater cognitive and behavioral burden compared to veteran with TBI only. Notably, epilepsy preceding TBI were found more often clustering in high behavioral risk profiles. This group with epilepsy preceding TBI was associated with, including dysfunction in the RDoC domains related to negative valence systems (44.4%), arousal/regulatory systems (37.0%), and interpersonal trauma.

**Discussion:**

These findings highlight the complex interplay between TBI and Epilepsy in shaping long term cognitive/behavioral challenges and point to the need for targeted clinical management, personalized treatment approaches, and refined therapeutic strategies to maximize the quality of life for affected veterans.

## Introduction

Epilepsy is a neurological condition commonly associated with a range of cognitive and behavioral impairments, including memory deficits, attentional difficulties, executive dysfunction, emotional disturbances, and social interaction problems (1–3). Historically, research primarily linked these impairments to damage or abnormalities in discrete brain regions; however, recent evidence emphasizes the importance of understanding epilepsy through network-based approaches that can model disruptions across interconnected neural circuits, rather than isolated areas (4). This integrative perspective acknowledges the complexity and heterogeneity of cognitive and behavioral symptoms, recognizing that they may exhibit diverse, complex symptom trajectories.

Traumatic brain injury (TBI) significantly increases the risk of developing epilepsy, and both conditions are reported more frequently among military personnel than the general population, with about a fifth of post-9/11 Veterans having history of TBI (5, 6). More than 80% of all military TBIs are mild in severity, and there is a statistically significant association between epilepsy and mild TBI (5, 6). People who experience both TBI and epilepsy often present with compounded neurobehavioral impairments, including heightened risk for anxiety, depression, post-traumatic stress disorder (PTSD), memory issues, executive dysfunction, and social integration challenges (7, 8). The interplay between TBI and epilepsy may further complicate these presentations, and a better understanding of these overlapping conditions is required to develop effective interventions.

To address the complexity inherent in these conditions, the Research Domain Criteria (RDoC) framework (9) offers an effective model for organizing symptoms across multiple dimensions: cognitive systems, emotional regulation (including negative and positive valence systems), social processing, sensorimotor functioning, and arousal/regulatory systems. Recent studies on cohorts with TBI and epilepsy have begun to explore phenotyping-based approaches to cluster individuals based on multiple correlated signs, symptoms, and diagnoses (10, 11). Extending upon these advances, the objective of this study was to identify the associations of distinct neurobehavioral phenotypes among veterans with epilepsy and TBI, including post traumatic epilepsy. By identifying more granular disease phenotypes that capture the diversity of epilepsy presentation, this work can help clinicians understand and predict symptom progression and inform the implementation of tailored interventions that target related underlying neural dysfunctions with greater effectiveness.

## Materials and methods

### Cohort development

The study cohort included post-9/11 veterans who entered Veteran Affairs (VA) health system to receive care between 2001 and 2012. Inclusion criteria required participants to have three or more years of VA care through the end of 2015 (including at least 1 year of care after 2007) and have data available in an existing VA data repository. To reduce heterogeneity introduced by varying TBI severity, we included only those with no history of TBI or Mild TBI. Using an epilepsy phenotype algorithm with a Positive predictive value of 0.92, we identified those with a diagnosis of epilepsy (12). Using dates of epilepsy diagnosis, we also identified those who developed epilepsy within 6 months after a TBI diagnosis. Lastly, we only included those with neuropsychological testing which we defined as having any, measured by the presence of at least one of the following CPT Codes (some having multiple): 96116, 96121, 96130, 96131, 96132, 96133, 96136, 96137, 96138, 96139, 96146. Per group, these criteria resulted in; Epilepsy (EPI) only: 11,317, TBI after EPI: 2,792, TBI before EPI: 5,646, TBI only: 137,479. We randomly sampled 200 individuals from each of these four groups to analyze their clinical text notes. Veterans with no history of TBI or epilepsy (677,105) were not included in further analysis.

### Statistical analysis

To compare sociodemographic and health characteristics across the four groups, we conducted group-level significance testing. Continuous variables such as age were analyzed using one-way Analysis of Variance (ANOVA). Categorical variables, including marital status, physical health conditions (e.g., seizures, headache, insomnia), and mental health diagnoses (e.g., anxiety, PTSD, depression), were assessed using Chi-square tests of independence. Variables with *p*-values < 0.05 were considered statistically significant. All analysis were performed using python 3.

### Moonstone ontology/grammar rule building

The Moonstone NLP platform is designed to extract useful features and derive variables from raw clinical text (13). Moonstone captures both explicit and implicit information by inferring complex concepts often embedded in the nuanced language of common narratives. Moonstone diverges from typical NLP systems that require unambiguous phrasing, as it was originally developed to recognize and extract subtle indicators from notes such as whether a patient lives alone.

The Moonstone NLP platform was trained to identify text features associated with cognitive and behavioral dysfunction. This involved the expansion of the searched ontologies to encompass new concepts and additional grammar rules including cognitive impairment, poor psychosocial function, and PTSD symptomatology, until the system achieved satisfactory accuracy against objective human rater labels of clinical notes.

To identify new rules and ontologies, human trainers selected best descriptions that Moonstone suggested when processing new text notes. Based on this input, new rule definitions were developed to identify cognitive and behavioral dysfunction in notes. The technique of expanding Moonstone’s capabilities was applied to sentences from a subset of reports to train the algorithm. A random forest classifier was used to predict cases with cognitive dysfunction using the trained Moonstone text features as inputs and evaluated on test data. The classifier demonstrated a high level of precision, accurately identifying cases with an 88% success rate, further confirming the supervised nature of the learning paradigm employed by Moonstone (14).

### Dimensional reduction

Uniform Manifold Approximation and Projection (UMAP) is a popular method for dimensionality reduction that accurately preserves the distance between observations in a high-dimensional symptom space when represented in a low-dimensional manifold (15). In this work, we embedded 39 features into two dimensions and tested for variability in results as a function of seed number; the results were found to be robust. Phenotypes were assigned by performing a nearest neighbor clustering of all individuals on their two-dimensional UMAP representation.

### RDOC domain

The Research Domain Criteria (RDoC) framework, developed by the National Institute of Mental Health (NIMH), guides research on mental disorders by focusing on fundamental dimensions of behavior and neurobiology rather than relying solely on traditional diagnostic categories (9). RDoC promotes an integrative, dimensional approach to understanding mental health that is conceptually well suited for phenotyping analyses. The framework identifies several major domains of human functioning including cognitive systems, negative and positive valence systems, social processes, arousal/regulatory systems, and sensorimotor systems.

We selected seven RDoC Domains for further analysis. Cognitive systems are responsible for various cognitive processes encompassing attention, perception, declarative memory, language, cognitive control and working memory. Negative Valence Systems are chiefly accountable for reactions to unpleasant situations, such as fear, anxiety, and loss. On the other hand, Positive Valence Systems are primarily responsible for responses to positive motivational scenarios, including seeking rewards, consummatory behavior, and learning from rewards and habits. Systems for Social Processes mediate responses in interpersonal settings, covering the perception and interpretation of others’ actions, one’s own actions, affiliation, and attachment. Arousal/Regulatory Systems are responsible for activating neural systems as needed for different contexts and maintaining appropriate homeostatic regulation of systems like energy balance and sleep. Lastly, Sensorimotor Systems are primarily tasked with controlling and executing motor behaviors, refining them through learning and development.

### Identification of cognitive and behavioral symptoms among study groups

We applied the Moonstone NLP software to each cohort to identify neurobehavioral phenotypes within the study population. We used the same text notes for manual chart review and NLP analysis. We organized the existing software ontology and created new ontologies as needed for this study (14). The main concepts were developed using RDoC (research Domain Criteria Initiative). The NLP ontology included 39 individual concepts (14). Each of concept was assigned to one RDoC category.

## Results

The sociodemographic and health characteristics of the four groups are summarized in Table 1. Each group included 200 individuals. Each group included 200 individuals. The mean age was similar across groups, with a mean (SD) of 45.9 (9.4) years in the EPI-only group, 46.3 (10.3) years in the EPI–TBI group, 45.3 (9.1) years in the TBI–EPI group, and 45.7 (9.8) years in the TBI-only group (*p* = 0.80). The majority of participants were White (66.5% EPI-only, 68.5% EPI–TBI, 74.0% TBI–EPI, and 66.5% TBI-only), followed by Black or African American participants (24.5%, 24.0%, 15.0%, and 22.0%, respectively), with smaller proportions identifying as other races (9.0%, 7.5%, 11.0%, and 12.0%). Gender distribution was identical across groups, with males comprising 50.0% of each cohort (*p* = 1.00). The proportion of married individuals differed across groups (*p* = 0.05), with 39.0% in the EPI-only group, 44.5% in the EPI–TBI group, 52.0% in the TBI–EPI group, and 40.0% in the TBI-only group, with the highest prevalence observed in the TBI–EPI group.

**Table 1:** Sociodemographic and health characteristics of veterans across study groups.

Characteristics	EPI only *N* = 200	EPI-TBI *N* = 200	TBI-EPI *N* = 200	TBI only *N* = 200	*p*-value
Sociodemographic characteristics					
Age (SD)	45.9 (9.4)	46.3 (10.3)	45.3 (9.1)	45.7 (9.8)	0.80
Race					
White	66.50%	68.50%	74.00%	66.50%	0.32
Black or African American	24.50%	24.00%	15.00%	22.00%	0.08
Others	9.00%	7.50%	11.00%	12.00%	0.43
Gender					
Male	50.00%	50.00%	50.00%	50.00%	1.00
Married					
Yes	39.00%	44.50%	52.00%	40.00%	0.05
Physical health characteristics					
Obesity	40.50%	33.50%	36.00%	39.50%	0.45
High blood pressure	35.50%	33.00%	32.00%	27.00%	0.32
Lung disease	18.00%	17.50%	19.00%	11.50%	0.17
Seizures	62.50%	62.50%	64.00%	0.00%	0.00
Obstructive sleep apnea	30.50%	29.00%	40.00%	29.00%	0.06
Headache	54.00%	74.00%	71.00%	51.50%	0.00
Insomnia	36.50%	43.50%	47.50%	35.50%	0.04
Mental health characteristics					
Anxiety	59.00%	59.50%	65.50%	50.00%	0.02
Suicide ideation	19.00%	26.00%	27.00%	12.50%	0.00
Bipolar	29.00%	37.00%	39.00%	18.50%	0.00
Post-Traumatic stress disorder	49.00%	69.50%	79.50%	67.50%	0.00
Depression	70.00%	75.00%	76.00%	66.00%	0.09
Substance use disorder	42.00%	49.50%	50.50%	40.00%	0.08

EPI Only, Epilepsy only; EPI-TBI, TBI after epilepsy; TBI-EPI, TBI before epilepsy; TBI Only, TBI without epilepsy.

Physical health conditions demonstrated notable group differences. Seizures were also varied significantly. Seizures were most common in the TBI before Epilepsy group (64%, *p* < 0.001). Headache prevalence differed by group (*p* < 0.001), peaking in the Epilepsy + TBI (74%) and the TBI before Epilepsy (71%), compared to Epilepsy Only (54%) and TBI Only (51.5%). Insomnia followed a similar pattern (*p* < 0.05), with higher rates in TBI before Epilepsy (47.5%) and Epilepsy + TBI (43.5%) groups.

Mental health characteristics showed multiple disparities. Anxiety was most frequent in the TBI before Epilepsy group (65.5%) compared to others (*p* < 0.05). Suicidal ideation also varied by groups (*p* < 0.00105), with the highest prevalence in TBI before Epilepsy (27%) and Epilepsy + TBI (26%) compared to 12.5% in TBI Only. Bipolar disorder followed a similar pattern (*p* < 0.001), with higher rates were elevated in TBI before Epilepsy (39%) and Epilepsy + TBI (37%) relative to TBI Only (18.5%) (*p* < 0.05). PTSD also showed strong differences (*p* < 0.001), with the prevalence was also highest in TBI before Epilepsy group (79.5%), followed by Epilepsy + TBI (69.5%), TBI Only (67.5%), and Epilepsy Only (49%) (*p* < 0.05).

To examine heterogeneity in neurobehavioral features, we extracted 39 ontology-based concepts from clinical notes using the Moonstone NLP platform (14) and represented each participant as a high-dimensional feature vector derived from the RDoC framework. We then applied UMAP to reduce this multidimensional space into two dimensions for visualization. In the resulting scatterplot, each of the 800 points corresponds to a representation of one veteran’s symptomology. Four distinct phenotypes emerged, each reflecting a different combination of cognitive and behavioral symptom domains. Two semi-independent dimensions captured much of the data variation: (1) symptom severity (low vs. high) and (2) symptom content (cognitive vs. behavioral).

**Figure 1:**
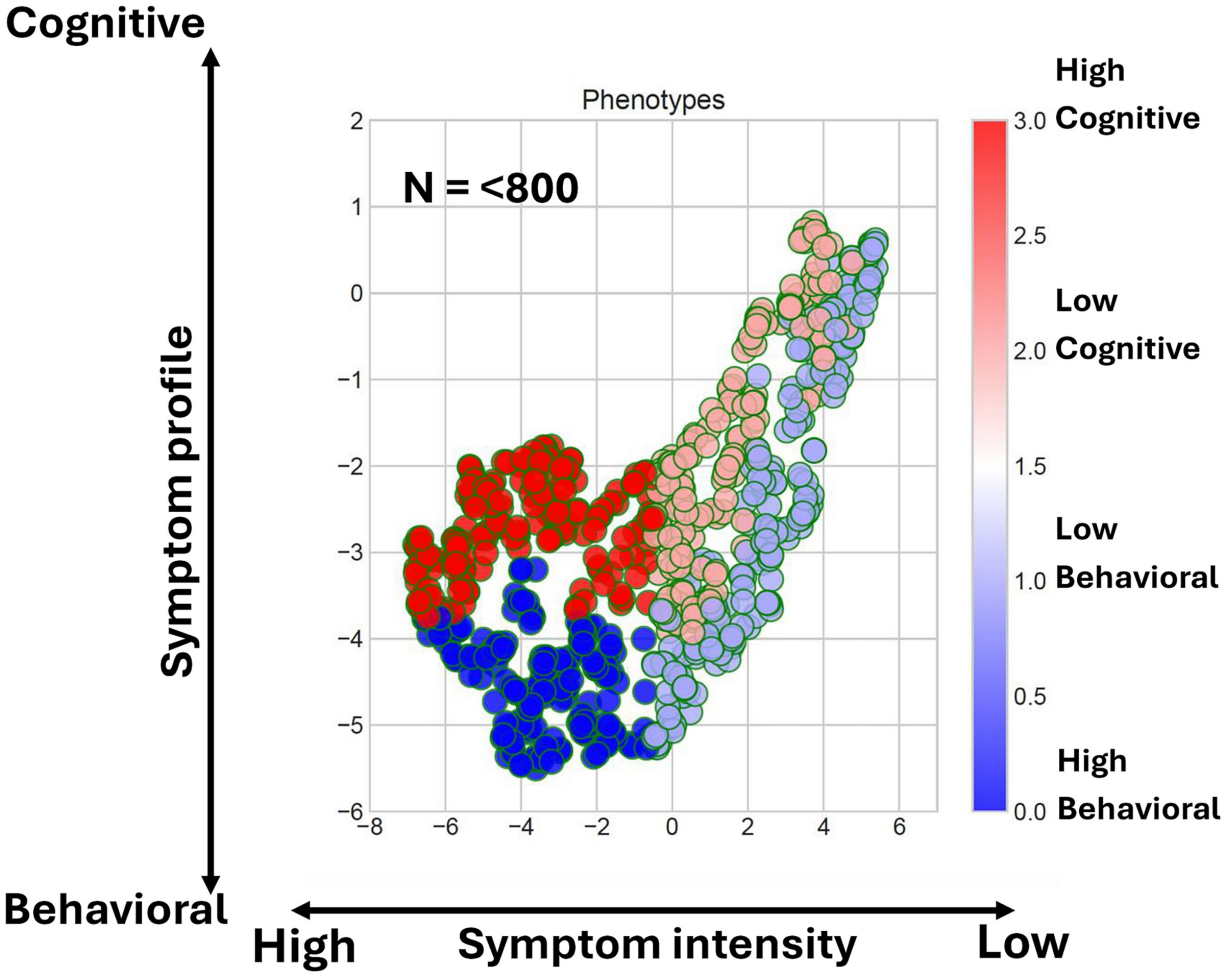
Two dimensional UMAP reduction of 39 NLP features for the full cohort, color coded by phenotypic membership.

In Figure 1, points that are close together indicate symptom profiles that are similar in the higher, 39-dimensional space. Color scale indicates phenotype assignment (Light Red = Low Cognitive Risk, Red = High Cognitive Risk, Cyan = Low Behavioral Risk, Blue = High Behavioral Risk). Clusters characterized by cognitive risk were distinguishable from those dominated by behavioral features, illustrating that neurobehavioral symptoms vary not only in intensity but also in composition. This approach demonstrates how dimensional reduction of seemingly complex, high dimensional data can yield straightforward, clinically actionable phenotypes.

**Figure 2:**
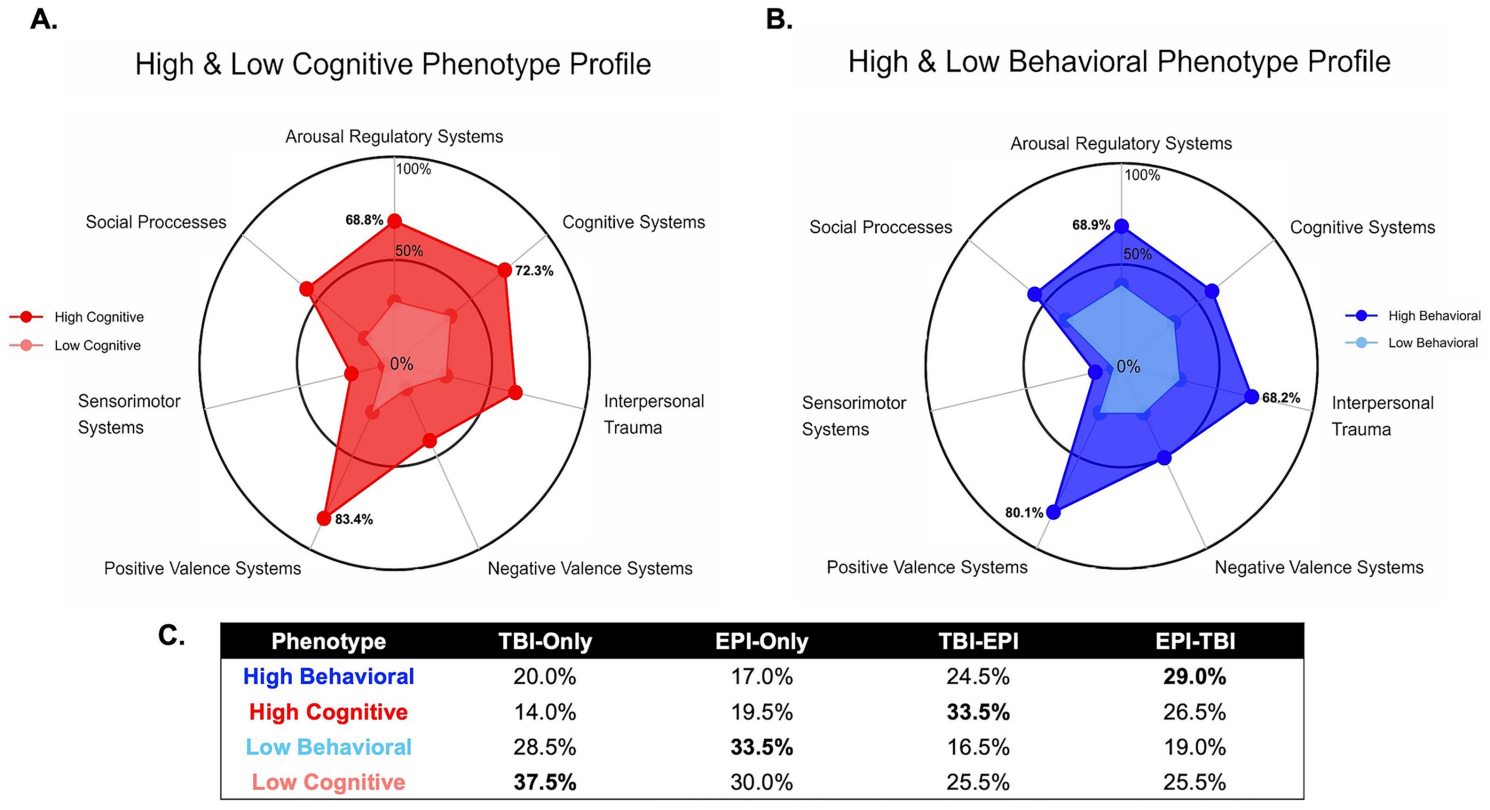
Profiling of neurobehavioral phenotypes. **(A)** Radar plot of the symptom profiles of the high/low symptom cognitive phenotypes. **(B)** Like **(A)**, but for behavioral phenotypes. **(C)** Distribution of phenotypes across TBI and epilepsy status.

Figure 2 illustrates the neurobehavioral phenotype profiles and their distribution across diagnostic groups. Figures 2A,B show radar plots to visualize relative impairment across RDoC domains for the cognitive and behavioral phenotypes, respectively. In Figure 2A, Veterans with a high cognitive phenotype demonstrated greater impairments in cognitive systems (72.3%) and arousal/regulatory systems (68.8%), whereas the low cognitive phenotype showed markedly lower dysfunction across these domains. In Figure 2B, the high behavioral phenotype was characterized by elevations in negative valence systems (50.3%), arousal/regulatory systems (68.9.0%), and interpersonal trauma (80.1%), in contrast to the low behavioral phenotype, which exhibited a lower and more evenly distributed symptom burden across RDoC domains.

Figure 2C displays the percentage distribution of each phenotype across the four diagnostic groups. A clear pattern emerged in the prevalences across TBI and epilepsy groups (bold values). The TBI-before-Epilepsy (TBI-EPI) group showed the highest prevalence of high cognitive phenotypes (33.5%) and a moderate prevalence of high behavioral risk (24.5%). The EPI-TBI group demonstrated the highest proportion of high behavioral phenotypes (29.0%), indicating a greater burden of behavioral dysfunction for those experience TBI events after epilepsy diagnosis. The Epilepsy-only group was most frequently represented in the low behavioral phenotype (33.5%), suggesting relatively fewer behavioral symptoms in this group. Finally, the TBI-only group showed the largest proportion of low cognitive phenotypes (37.5%), suggesting lower cognitive risk compared to the other groups.

Together, these findings indicate that while both cognitive and behavioral phenotypes were present across all groups, with TBI-EPI veterans more often clustering in high cognitive risk profiles and EPI-TBI veterans more often clustering in high behavioral risk profiles.

## Discussion

In this study, we identified four neurobehavioral phenotypes among veterans with TBI and epilepsy. Data was drawn from clinical notes and analyzed using a combined NLP and clustering approach that was based on an existing RDoC ontology. The phenotypes primarily differentiated along two axes: severity of dysfunction (low vs. high) and symptom type (cognitive vs. behavioral). Veterans with TBI before epilepsy were most frequently assigned to high cognitive risk profiles, whereas those with epilepsy before TBI were more often classified in high behavioral profiles. Epilepsy-only veterans were more often represented in lower-risk behavioral phenotypes, while TBI-only veterans showed higher proportions in lower-risk cognitive phenotypes. Taken together, these findings suggest diverging trajectories that depend on the temporal sequence of TBI and Epilepsy.

The concentration of cognitive impairment in the TBI-EPI group is consistent with recent population-based evidence linking posttraumatic epilepsy (PTE) to poor long-term neurological outcomes (16). This large cohort study found that individuals with PTE had a 4.5-fold higher risk of developing dementia compared with individuals without a history of head injury or epilepsy (16). Longitudinal evidence also shows that TBI alone is linked to accelerated cognitive decline (17). When seizures develop after TBI, they may act as an additional “second hit.” This interpretation is also supported by a systematic review of clinical studies, which reported consistently poorer cognitive performance in individuals with PTE compared with those with TBI alone (18). In addition, a cohort study also demonstrated higher prevalence of cognitive impairment in TBI patients who developed PTE later compared with those without seizures (19). This combination likely explains why the TBI-EPI group in our cohort clustered in high cognitive risk phenotypes; epilepsy amplifies the cognitive burden of TBI and accelerates decline.

In contrast, veterans with epilepsy before TBI more often exhibited behavioral dominant phenotypes, consistent with the well-established psychiatric burden of epilepsy. Depression and anxiety affect up to 30%–40% of people with epilepsy, bipolar disorder occurs in approximately 12%, and the risk of suicidality is at least doubled compared to the general population (20). Some risk for TBI events may also be attributed to behavioral changes. In parallel, TBI may worsen epilepsy related behavioral changes by further disrupting emotion regulation, sleep, and stress (21).

### Limitations

This study has strengths and limitations. Our study relied on an NLP-derived symptom extraction platform that outperforms conventional NLP at clinical text analysis tasks. However, patient underreport and clinician under-documentation are common in clinical text data, and symptoms may have been underreported for certain conditions. Although the sample size (*N* = 200 each) moderately limited statistical power, group differences were strong and significant. We restricted analysis to veterans with mild TBI, because the association between mild TBI and epilepsy related symptoms has not received significant research attention. However, this inclusion criteria may limit the applicability of our findings to veterans with more severe TBI severity.

Future work should track veterans longitudinally to assess whether patients transition between phenotypes and should assess the impact and role of TBI severity and epilepsy severity (e.g., seizure intensity and frequency) on the prevalence of phenotypes. Prospective studies should also incorporate structured neuropsychological testing and psychiatric assessments. Future interventions could assess whether phenotype membership is a useful parameter for guiding effective treatment. Finally, extending analyses to incorporate biomarkers and/or imaging would help clarify the mechanism and biological basis of the cognitive and behavioral phenotypes identified, thereby strengthening clinical translation.

## Conclusion

This study advances our understanding of neurobehavioral heterogeneity in veterans with epilepsy and TBI by identifying distinct phenotypic clusters that transcend traditional diagnostic boundaries. Severity and symptom profiles varied within traditional clinical categories in meaningful ways. Our findings have important implications for risk stratification and treatment planning. By leveraging advanced computational approaches to parse clinical complexity, this work provides a foundation for developing more precise, personalized approaches to managing the complex neurobehavioral sequelae of epilepsy and TBI in veterans. These findings emphasize the critical need to more deeply explore integrated, phenotype-informed care models that could help address longstanding issues among veterans living with TBI and epilepsy.

## Data Availability

The data analyzed in this study are subject to the following restrictions: the datasets are part of the U.S. Department of Veterans Affairs (VA) medical record system and are not publicly available. Requests for access to these data should be directed to maryjo.pugh@hsc.utah.edu. Access may be granted upon approval and completion of applicable VA security and privacy requirements.
